# BCG vaccination induces enhanced frequencies of memory T cells and altered plasma levels of common γc cytokines in elderly individuals

**DOI:** 10.1371/journal.pone.0258743

**Published:** 2021-11-10

**Authors:** Nathella Pavan Kumar, Chandrasekaran Padmapriyadarsini, Anuradha Rajamanickam, Perumal Kannabiran Bhavani, Arul Nancy, Bharathi Jayadeepa, Nandhini Selvaraj, Dinesh Asokan, Rachel Mariam Renji, Vijayalakshmi Venkataramani, Srikanth Tripathy, Subash Babu

**Affiliations:** 1 ICMR-National Institute for Research in Tuberculosis-International Center for Excellence in Research, Chennai, India; 2 ICMR-National Institute for Research in Tuberculosis, Chennai, India; 3 Dr D Y Patil Medical College, Hospital and Research Centre, Pune, India; Centenary Institute, AUSTRALIA

## Abstract

BCG vaccination is known to induce innate immune memory, which confers protection against heterologous infections. However, the effect of BCG vaccination on the conventional adaptive immune cells subsets is not well characterized. We investigated the impact of BCG vaccination on the frequencies of T cell subsets and common gamma c (γc) cytokines in a group of healthy elderly individuals (age 60–80 years) at one month post vaccination as part of our clinical study to examine the effect of BCG on COVID-19. Our results demonstrate that BCG vaccination induced enhanced frequencies of central (p<0.0001) and effector memory (p<0.0001) CD4+ T cells and diminished frequencies of naïve (p<0.0001), transitional memory (p<0.0001), stem cell memory (p = 0.0001) CD4+ T cells and regulatory T cells. In addition, BCG vaccination induced enhanced frequencies of central (p = 0.0008), effector (p<0.0001) and terminal effector memory (p<0.0001) CD8+ T cells and diminished frequencies of naïve (p<0.0001), transitional memory (p<0.0001) and stem cell memory (p = 0.0034) CD8+T cells. BCG vaccination also induced enhanced plasma levels of IL-7 (p<0.0001) and IL-15 (p = 0.0020) but diminished levels of IL-2 (p = 0.0033) and IL-21 (p = 0.0020). Thus, BCG vaccination was associated with enhanced memory T cell subsets as well as memory enhancing γc cytokines in elderly individuals, suggesting its ability to induce non-specific adaptive immune responses.

## Introduction

Bacillus Calmette-Guerin (BCG) is a live—attenuated vaccine strain of *Mycobacterium bovis* that provides protection against mycobacterial infections such as tuberculosis and leprosy and was first introduced in 1921 [1, 2]. In addition to protective immunity to mycobacterial infections, BCG is also known to protect against heterologous infections (the so-called off-target or non—specific effects) [3, 4]. Several epidemiological studies have shown a reduction in childhood mortality in BCG vaccinated children as well as lower incidence of respiratory infections [5–9]. In addition, randomized controlled trials have shown that BCG vaccination protects against childhood mortality mainly by providing protection against neonatal sepsis and respiratory infections [10–12]. Meta-analysis studies have shown that BCG protects against *Mycobacterium tuberculosis* infection as well as progression from infection to disease for up to 10 years of age [13–15]. The effect of BCG vaccination in protecting against heterologous infections in adults and more specifically, elderly individuals is less well studied.

Two types of immune mechanisms have been postulated to explain this off-target or non—specific effect of BCG against infections. First, BCG is known to induce CD4^+^ and CD8^+^ memory T cells in an antigen-independent but cytokine-dependent manner, and this process is termed heterologous immunity [16–19]. Second, BCG is known to induce a process called trained immunity or innate immune memory in innate cells, especially monocytes and NK cells, such that these cells can respond more actively to secondary or bystander infections [20, 21]. However, whether these mechanisms are operational in elderly individuals, who are at higher risk for infections due to waning immunity is not known.

One principal family of type I cytokines is the common γc family, which consists of IL-2, IL-7, IL-15 and IL-21 [22] and these cytokines are effective growth factors for T cells [23]. Published studies have reported that γc cytokines contribute to the induction of T cell responses and also it has a vital role in the development or maintenance of memory T cells [24, 25], but the association between memory T cell subsets and these common γc cytokines in BCG vaccinated elderly individuals has not been examined. Hence, we examined the induction of T cell subsets and circulating γc cytokines levels in response to BCG vaccination in elderly individuals at baseline and one-month post-vaccination along with baseline frequencies in unvaccinated individuals. We demonstrate that BCG vaccination induces significantly enhanced memory T cell responses and altered γc cytokines, suggesting that BCG can potentially boost immune responses in a non -specific or off-target manner in these elderly individuals.

## Materials and methods

### Ethics statement

The study was approved by the Ethics Committees of NIRT (NIRT-INo:2020010). Informed written consent was obtained from all participants. The study is part of the clinical study entitled, Study to evaluate the effectiveness of the BCG vaccine in reducing morbidity and mortality in elderly individuals in COVID-19 hotspots in India (NCT04475302).

### Study population

Elderly individuals, between 60–80 years of age, residing in hotspots for SARS-Cov2 infection were included in the study between July 2020 and September 2020 in Chennai, India after obtaining informed written consent from the study participants. Elderly population positive for SARS-Cov2 infection by either antibody (serology) or PCR test; HIV infected or individuals with malignancy or on immunosuppressive drugs or transplant recipient and those on dialysis or anti-psychiatric medications or hypersensitivity to vaccinations were not included in the study. Also, those who were diagnosed with tuberculosis (TB) in the previous 6-months or were currently on anti-TB treatment were not included in the study. Latent TB screening was done by interferon gamma release assay (IGRA) test. 54 participants received a single dose of BCG vaccine (Freeze-dried) manufactured by Serum Institute of India, Pune. The adult dose of BCG vaccine was 0.1 mL injected intradermally over the distal insertion of the deltoid muscle onto the left humerus (approximately one third down the left upper arm). In case of a previous vaccination scar, or presence of ulcer/injury or tattoo on the left upper arm, vaccination was given in the right upper arm. 32 elderly individuals from the same hotspot area were not vaccinated and were considered as controls. Blood was drawn from the vaccinated participants at baseline (before vaccination) and 1 month following vaccination. Blood was drawn from the controls only at baseline. The demographic and epidemiological data have been previously reported [26].

### Ex vivo analysis

All antibodies used in the study were from BD Biosciences (San Jose, CA), BD Pharmingen (San Diego, CA), eBioscience (San Diego, CA), or R&D Systems (Minneapolis, MN). Whole blood was used for ex vivo phenotyping and it was performed on all 86 individuals. Briefly, to 250μl aliquots of whole blood a cocktail of monoclonal antibodies specific for various immune cell types was added. T cell phenotyping was performed using antibodies directed against CD45 Peridinin chlorophyll protein (PerCP), CD3 phycoerythrin (PE) Cy7, CD4 allophycocyanin-H7 (APC-H7), CD8 AmCyan, CD28 APC, CD45RA Pacific Blue, CCR7-FITC and CD95 PE. Naive cells were classified as CD45RA^+^ CCR7^+^ CD95^-^ CD28^+^, central memory cells (T_CM_) as CD45RA^-^ CCR7^+^ CD95^+^ CD28^+^, effector memory cells (T_EM_) as CD45RA^-^CCR7^-^ CD95^+^ CD28, Terminal effector memory cells (T_TEM_) as CD45RA^-^ CCR7^-^ CD95^+^ CD28^-^, stem cell memory (T_SCM_) as CD45RA^+^ CCR7^+^ CD95^+^ CD28^+^ and transitional memory cells (T_TM_) as CD45RA^+^ CCR7^-^ CD95^+^ CD28^+^ [27]. Regulatory T cell phenotyping was performed using CD3 Amycyn, CD4 APC-H7, CD25 APC, CD127 FITC, Foxp3 PE and regulatory T cells were classified as CD4^+^ CD25^+^ Foxp3^+^ CD127dim [27]. Following 30 min of incubation at room temperature erythrocytes were lysed using 2 ml of FACS lysing solution (BD Biosciences Pharmingen), and cells were washed twice with 2 ml of 1XPBS and suspended in 200 μl of PBS (Lonza, Walkersville, MD). Eight- color flow cytometry was performed on a FACS Canto II flow cytometer with FACSDIVA software, version 6 (Becton Dickinson). The gating was set by forward and side scatter, and 100 000 gated events were acquired. Data were collected and analyzed using FLOW JO software 10.7.1 (TreeStar, Ashland, OR). Leukocytes were gated using CD45 expression versus side scatter [28, 29].

### ELISA

Circulating levels of IL-2, IL-7 and IL-15 were measured using Luminex Human Magnetic multiplex assay kit (R&D Systems). IL-21 levels were measured using the IL-21 Human ELISA kit (Invitrogen). The lowest detection limits were as follows: IL-2, 3.6 pg/mL; IL-7, 3.5 pg/mL; IL-15, 2.5 pg/mL; and IL-21, 78 pg/mL. The lowest standard value was assigned to the samples that were below the threshold of detection.

### Statistical analysis

Geometric means (GM) were used for measurements of central tendency. Wilcoxon signed-rank test was used to compare frequencies of immune subsets and γc cytokines in the BCG vaccinated group at month 0 (M0) and month 1 (M1). Statistically significant differences between unvaccinated and BCG vaccinated M1 groups were analyzed using the Mann-Whitney test. Analyses were performed using Graph-Pad PRISM Version 9.0. Correlation matrix analysis was done using statistical software JMP 14.0 (SAS, Cary, NC, USA).

## Results

### Study population

The demographics of the study population are shown in Table 1. From July 2020 through September 2020, 86 individuals were enrolled in the study with 54 in the vaccinated arm and 32 in the unvaccinated arm. All the vaccinated individuals were followed up at month 1 post-vaccination with no loss to follow-up. Median age was 65 (Range: 60–78) years in BCG vaccinated group and 63 years (Range: 60–80) in the unvaccinated group. There were 34 males and 20 females in the BCG vaccinated and 15 males and 17 females in unvaccinated group. In the enrolled population, 26% of BCG vaccinated and 15% of unvaccinated individuals had diabetes mellitus while 15% and 9% had cardiovascular disease respectively. In our cohort, 4%-6% were current smokers and there were 6% were alcoholics. Other baseline characteristics were similar between the two arms.

**Table 1:** Demographics of the study population.

	Vaccinated		Non- Vaccinated	P Value
Subjects Enrolled	n = 54		n = 32	
	Month 0 (n = 54)	Month 1 (n = 54)		
Age (Median)	65 (60–78)		63 (60–80)	p = 0.7333
Gender (M/F)	34/20		15/17	
Height (Median)	160 cm		155 cm	p = 0.5684
Weight (Median)	62 Kg		63 Kg	p = 0.5321
Pulse rate (Median)	86		88	p = 0.4422
Systolic Blood Pressure (Median)	132		140	p = 0.3211
Diastolic Blood Pressure (Median)	81		80	p = 0.5322
SPOS% (Median)	98		98	p = 0.9432
Diabetes Mellitus no. (%)	14 (26%)		5 (15%)	p = 0.0743
Smoking, no. (%)	2 (4%)		2 (6%)	p = 0.8633
Alcoholism, no. (%)	3 (6%)		2 (6%)	p = 0.8239
Cardiovascular Disease, no. (%)	8 (15%)		3 (9%)	p = 0.0888
Respiratory Diseases, no. (%)	5 (9%)		2 (6%)	p = 0.4521
Interferon gamma release assay (IGRA)—Postive	27 (50%)		16 (50%)	p = 0.5321
BCG Scar—Yes	5 (9%)		3 (9%)	p = 0.6321

BCG vaccination induces enhanced frequencies of central and effector memory CD4^+^ T cells and diminished frequencies of naïve, transitional, and stem cell memory CD4^+^ T cells

### BCG vaccination induces enhanced frequencies of central and effector memory CD4^+^ T cells and diminished frequencies of naïve, transitional, and stem cell memory CD4^+^ T cells

**Fig 1:**
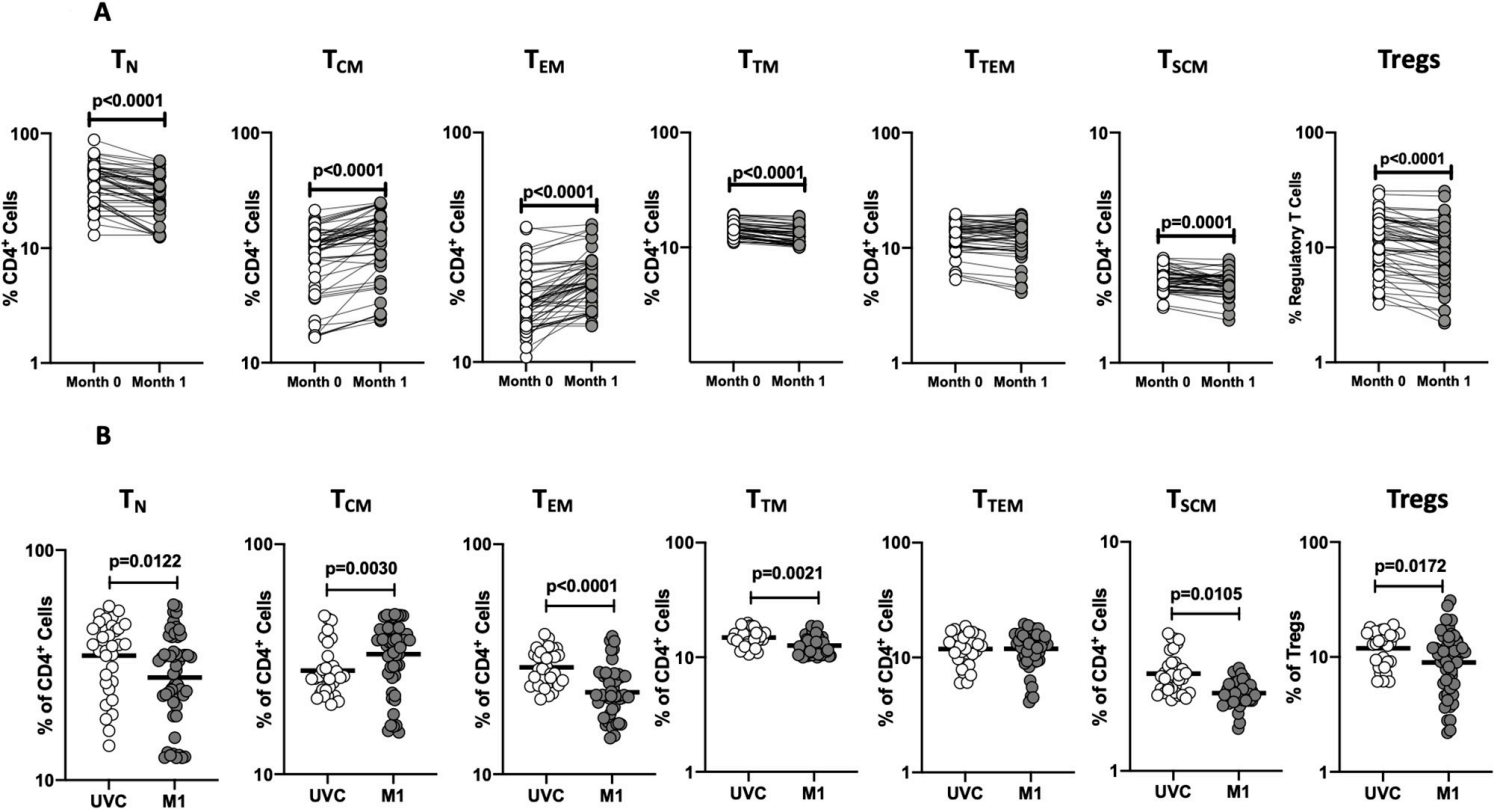
BCG vaccination is associated with altered frequencies of CD4^+^ T cell memory subsets and regulatory cells. **(A)** Frequencies of CD4^+^ T cell subsets in BCG pre-vaccinated [M0] (n = 54) and month 1 following vaccination [M1] (n = 54). Data are shown as line diagrams with each line representing a single individual. p values were calculated using the Wilcoxon matched pair tests with Holms correction for multiple comparisons. **(B)** Frequencies of CD4^+^ T cell subsets in BCG unvaccinated (UVC) (n = 32) and post vaccinated [M1] (n = 54) individuals. The data are represented as scatter plots with each circle representing a single individual. p values were calculated using the Mann-Whitney test with Holm’s correction for multiple comparisons.

To assess the ex vivo phenotype of CD4^+^ T cell subsets following BCG vaccination, we compared the subsets at baseline or before BCG vaccination (M0) and at month 1 (M1) post-vaccination. A representative flow cytometry plot showing the gating strategy for CD4^+^ T cell subsets of 0 month is shown in S1 Fig. As shown in Fig 1A, the frequencies of central and effector memory CD4^+^ T cell subsets were significantly enhanced and the frequencies of naïve, transitional, and stem cell memory CD4^+^ T cells and regulatory CD4^+^ T cells were significantly diminished at M1 compared to M0. Next, we compared the frequencies of CD4^+^ T cell subsets in post-vaccinated individuals to unvaccinated controls. As shown in Fig 1B, BCG vaccinated individuals exhibited increased frequencies of only central memory CD4^+^ T cells and decreased frequencies of naïve, effector memory, transitional memory, stem cell memory, and regulatory CD4^+^ T cells. Thus, BCG vaccination induces enhanced frequencies of central and effector memory CD4^+^ T cells in elderly individuals.

### BCG vaccination induces enhanced frequencies of central, effector, and terminal effector memory CD8^+^ T cells and diminished frequencies of naïve, transitional, and stem cell memory CD8^+^ T cells

**Fig 2:**
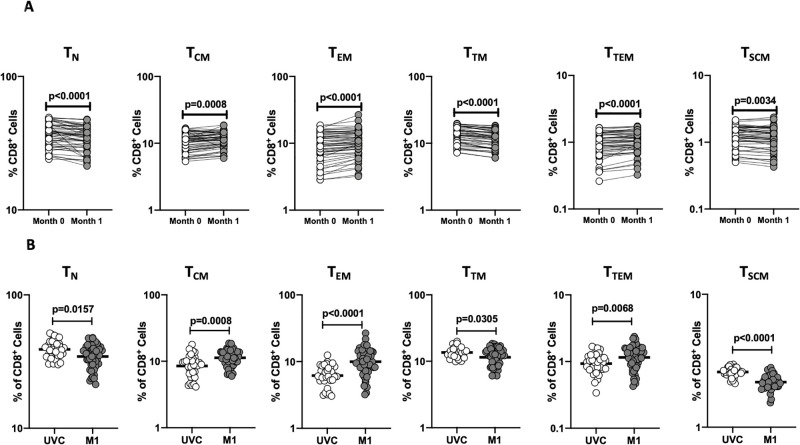
BCG vaccination is associated with altered frequencies of CD8^+^ T cell memory subsets. **(A)** Frequencies of CD8^+^ T cell subsets in BCG pre-vaccinated [M0] (n = 54) and month 1 following vaccination [M1] (n = 54). Data are shown as line diagrams with each line representing a single individual. p values were calculated using the Wilcoxon matched pair tests with Holms correction for multiple comparisons. **(B)** Frequencies of CD8^+^ T cell subsets in BCG unvaccinated (UVC) (n = 32) and post vaccinated [M1] (n = 54) individuals. The data are represented as scatter plots with each circle representing a single individual. p values were calculated using the Mann-Whitney test with Holm’s correction for multiple comparisons.

To assess the ex vivo phenotype of CD8^+^ T cell subsets following BCG vaccination, we compared the subsets at baseline or before BCG vaccination (M0) and at month 1 (M1) post-vaccination. A representative flow cytometry plot showing the gating strategy for CD8^+^ T cell subsets of 0 month is shown in S1 Fig. As shown in Fig 2A, the frequencies of central, effector, and terminal effector memory CD8^+^ T cell subsets were significantly enhanced and the frequencies of naïve, transitional and stem cell memory CD8^+^ T cells were significantly diminished at M1 compared to M0. Next, we compared the frequencies of CD8^+^ T cell subsets in post-vaccinated individuals to unvaccinated controls. As shown in Fig 2B, BCG vaccinated individuals exhibited increased frequencies of central memory, effector memory, and terminal effector memory CD8^+^ T cells and decreased frequencies of naïve, transitional memory, and stem cell memory CD8^+^ T cells. Thus, BCG vaccination induces enhanced frequencies of central, effector, and terminal effector memory CD8^+^ T cells in elderly individuals.

### BCG vaccination induces enhanced plasma levels of IL-7 and IL-15 and diminished plasma levels IL-2 and IL-21 levels

**Fig 3:**
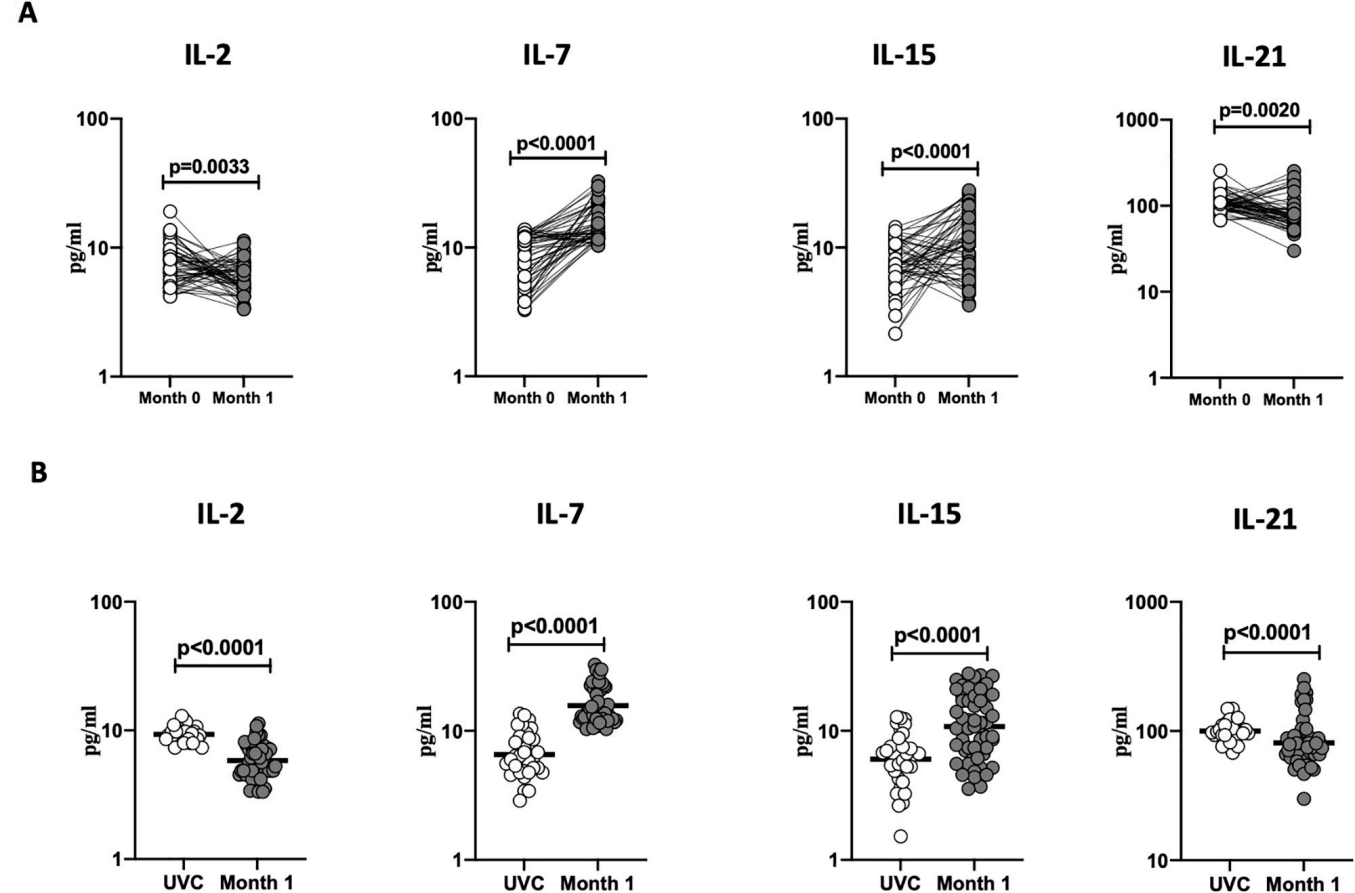
BCG vaccination is associated with altered circulating levels of γc cytokines. (A) The plasma levels of common γc cytokines IL-2, IL-7, IL-15 and IL-21were measured in BCG pre-vaccinated [M0] (n = 54) and month 1 following vaccination [M1] (n = 54). Data are shown as line diagrams with each line representing a single individual. p values were calculated using the Wilcoxon matched pair tests with Holms correction for multiple comparisons. **(B)** The plasma levels of common γc cytokines IL-2, IL-7, IL-15 and IL-21in BCG unvaccinated (UVC) (n = 32) and post vaccinated [M1] (n = 54) individuals. The data are represented as scatter plots with each circle representing a single individual. p values were calculated using the Mann-Whitney test with Holm’s correction for multiple comparisons.

To examine the plasma levels of common γc cytokines following BCG vaccination, we compared the plasma levels of IL-2, IL-7, IL-15 and IL-21 at baseline or before BCG vaccination (M0) and at month 1 (M1) post-vaccination. As shown in Fig 3A IL-2 (p = 0.033), IL-21 (p = 0.0020) showed significantly diminished levels at M1 compared to M0, however the plasma levels of IL-7 (p<0.0001) and IL-15 (p<0.0001) were significantly increased M1 compared to M0. Next, we compared the plasma levels of γc cytokines in post-vaccinated individuals to unvaccinated controls. As shown in Fig 3B BCG vaccinated individuals exhibited decreased circulating level of IL-2 (p<0.0001) and IL-21 (p<0.0001) however circulating levels of IL-7 (p<0.0001) and IL-15 (p<0.0001) were increased. Thus, BCG vaccination induces altered systemic levels in elderly individuals.

### Associations between memory T cells markers and γc cytokines

**Fig 4:**
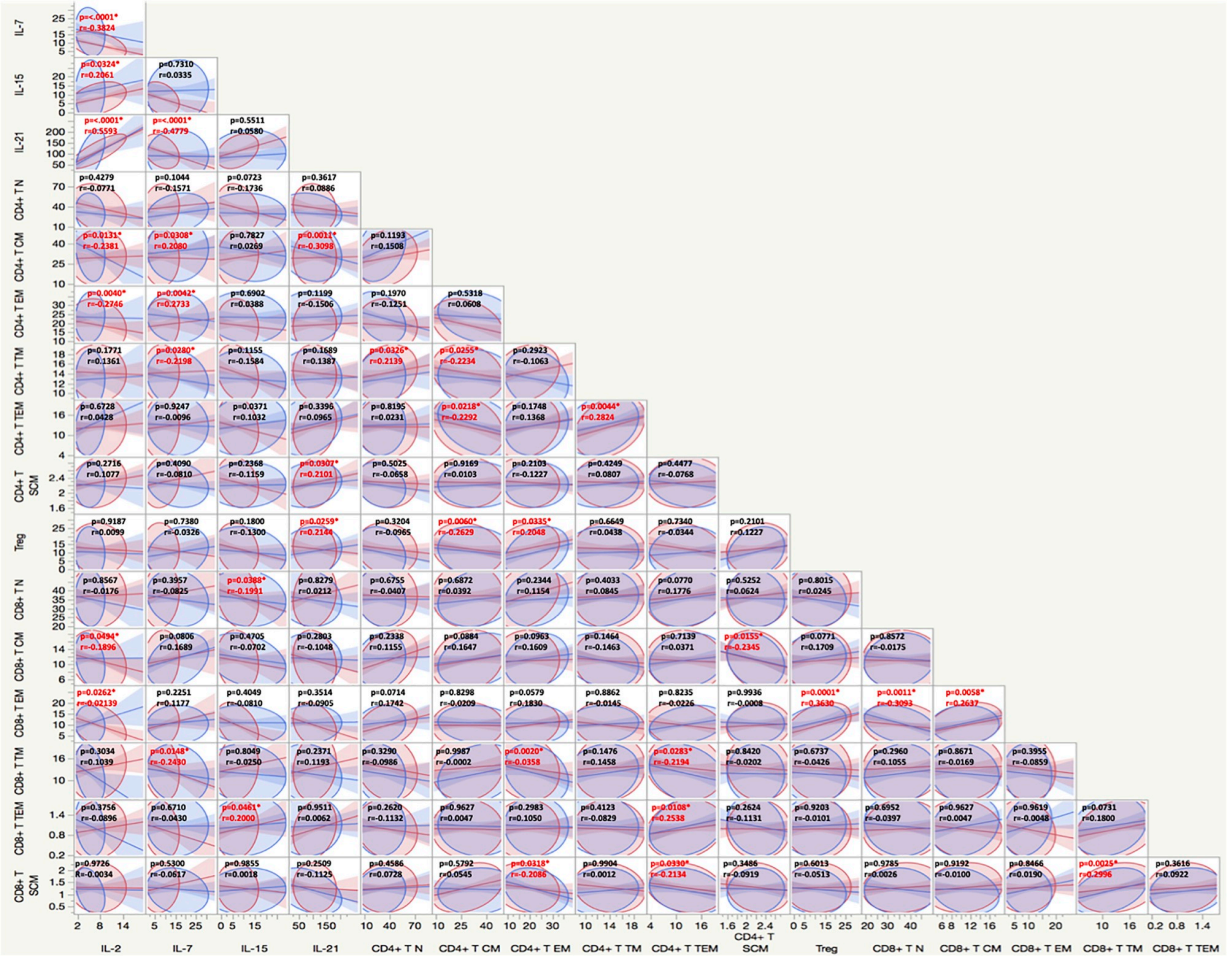
Relationship between CD4+ and CD8+ memory T cell subsets and γc cytokines. Multiparametric matrix correlation plot of CD4+ and CD8+ memory T cell subsets, and γc cytokines in all individuals with BCG pre-vaccinated and month 1 following vaccination. Spearman’s correlation coefficients are visualized. The blue line represents the x-axis parameter, and the red line represents the y-axis parameter.

We wanted to identify correlations between frequencies of CD4+ and CD8+ memory T cells subsets and γc cytokines in BCG vaccinated individuals. As shown in Fig 4A, multiparametric matrix correlation plot showed strong positive correlations between plasma levels of IL-7 with the frequencies of central memory (p = 0.0308), effector memory (p = 0.0042) and translational memory (p = 0.0280) cells and IL-15 was positively correlated with the naïve cells (p = 0.0388) and terminal effector memory T cells (p = 0.0461), whereas the circulating levels of IL-2 was negatively correlated with frequencies of central (p = 0.0131) and effector memory T cells (p = 0.0040) and finally IL-21 was negatively correlated with central memory (p = 0.0011) cells but positively correlated with stem cell like memory (p = 0.0307) and regulatory T cells (p = 0.0259). Our results overall indicate an association between the memory T cells subsets and γc cytokines.

## Discussion

Typically, elderly individuals are at high risk of infectious diseases. In the context of COVID-19 disease, elderly individuals are one of the main target groups for morbidity and mortality. Several clinical trials are currently underway to examine the effect of BCG vaccination in protecting health care workers and other individuals from SARS-CoV2 infection or disease [30–33]. Very few studies are examining the protective effect of BCG vaccination against SARS-CoV2 in elderly individuals [32]. Other published studies from India have also reported that BCG revaccination of young adults can boost a Mtb-specific CD4^+^ T cell immune responses possibly related with controlled TB infection [34]. The study to evaluate the effectiveness of the BCG vaccine in reducing morbidity and mortality in elderly individuals in COVID-19 hotspots in India undertaken by the Indian Council of Medical Research is one such study. As part of the study protocol, we examined the immune responses engendered by BCG vaccination in a group of elderly individuals. Previous studies in elderly individuals have shown that BCG vaccination protected against respiratory infections in Indonesia, Japan, and Europe [35–37].

Memory T cells are important in providing vaccine-induced protection against infections in elderly individuals [38]. Memory CD4^+^ T cells induced by vaccination, especially central and effector memory CD4+ T cells, are involved in preventing varicella-zoster reactivation in older individuals [39, 40]. Memory CD8^+^ T cells induced by vaccination, mainly central and effector memory CD8^+^ T cells, are involved in providing immunity against viral pathogens such as influenza and respiratory syncytial virus [41–44]. While other CD4^+^ and CD8^+^ memory T cell subsets, including transitional memory, terminal effector memory, and stem cell memory subsets, have been postulated to play a role in infection and cancer [45–47], their exact role in vaccine-elicited human immune responses is still unclear. However, induction of vaccine-induced T cell responses is clearly impaired in elderly individuals [38]. One potential mechanism is the expansion of regulatory CD4^+^ T cells, which are known to modulate effector T cell responses against pathogens [48].

Our study clearly demonstrates the increased frequencies of both CD4^+^ and CD8^+^ central and effector memory T cells. In addition, terminal effector CD8^+^ T cell memory frequencies are also increased. This is associated with a decrease in the other CD4^+^ and CD8^+^ T cell subsets. Therefore, the heightened frequency of the central and effector memory T cell compartment signifies a potential impact on the heterologous immunity and suggests that non-specific or bystander infections are more likely to be protected against in BCG vaccinated individuals. These data fit very well with the recent finding that BCG vaccination in elderly patients was associated with increased time to first infection and protection against viral respiratory pathogens [35]. Moreover, BCG vaccination also diminishes the frequency of regulatory T cells and could therefore potentially blunt or ameliorate the regulatory effects of these cells in down modulating protective immune responses.

Common γc cytokines are important for the generation and peripheral homeostasis of T cells, and the levels of γc cytokine are differentially modulated during immune response [9] Among the γc cytokines, IL-2, IL-7, IL-15 and IL-21 offer varied functions related to T-cell survival, activation and clonal expansion, and memory-cell development and maintenance, more specifically IL-7 which facilitates the survival of naive and memory T cells [49, 50] and IL-15 which is also essential for the homeostatic proliferation of memory CD8+ T cells and conservation of the steady-state level of CD8+ T-cell memory response [51, 52]. In addition, studies [53] have also reported that IL-15 might exert direct effects on memory T cell responses. To support these findings, our data also clearly reveal that increased effector memory and central memory T cell subsets are associated with increases in both IL-7 and IL-15. This suggests that IL-7 and IL-15 might potentially synergize to promote the generation of memory T cells responses in BCG vaccinated individuals.

The other γc cytokine, IL-2 is mostly produced by T cells, mainly the CD4+ Th1 subsets and also by activated CD8+ T cells [54]. Our current findings suggest that upon one month of post BCG vaccination, IL-2 levels are significantly diminished and thus unlikely to contribute to enhanced memory T cell frequencies. Like IL-2, the other γc cytokine IL-21 also has a role on both innate and adaptive immune responses [55] and IL-21 can also stimulate the activation, proliferation and differentiation of T cells [56]. Our study reveals that IL-21 is significantly diminished post vaccination and therefore also be unlikely to influence the memory T cell response.

In summary, our study highlights the effect of BCG vaccination in modulating the frequencies of adaptive immune cell subsets. Study limitations are that samples were collected only during the baseline visit and not at follow up in control individuals and that all the measured data are reported only in percentages but not absolute numbers. Our study also reveals an effect of BCG in inducing good correlation with γc cytokines and memory T cells subsets. Although our study did not examine the functional effects of these changes in the immune system, our data nevertheless reveal an important role for BCG vaccination in boosting immune responses in the elderly population. Whether this translates to improved protective immunity to non—specific infections like SARS-CoV2 remains to be determined.

## Supporting information

S1 Fig Gating strategy for CD4^+^ and CD8^+^ cell subsets and regulatory T cells. A representative pseudocolur flow cytometry plot from an BCG vaccinated individuals.
(TIFF)

S1 Table Names, sources, dilutions, and catalog numbers for each antibody used in this study. (TIFF)
