# Supplementary figures and images for: BCG vaccination induces enhanced frequencies of memory T cells and altered plasma levels of common γc cytokines in elderly individuals
Source: PLoS One. 2021 Nov 10;16(11):e0258743. doi: 10.1371/journal.pone.0258743 (PMC8580239; doi:10.1371/journal.pone.0258743)

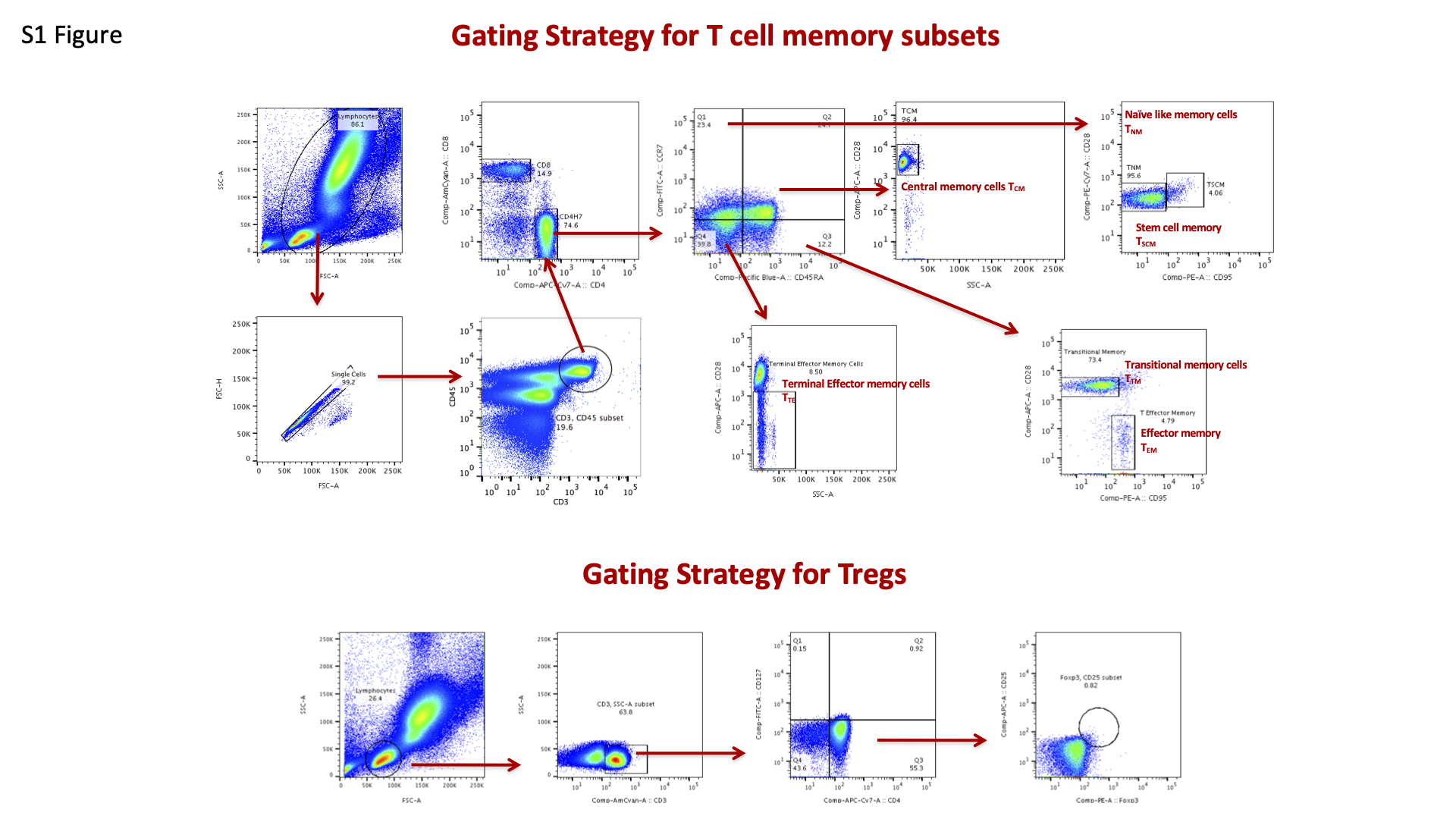

Supplement: S1 Fig — A representative pseudocolur flow cytometry plot from an BCG vaccinated individuals. (TIFF) [file pone.0258743.s001.tiff]

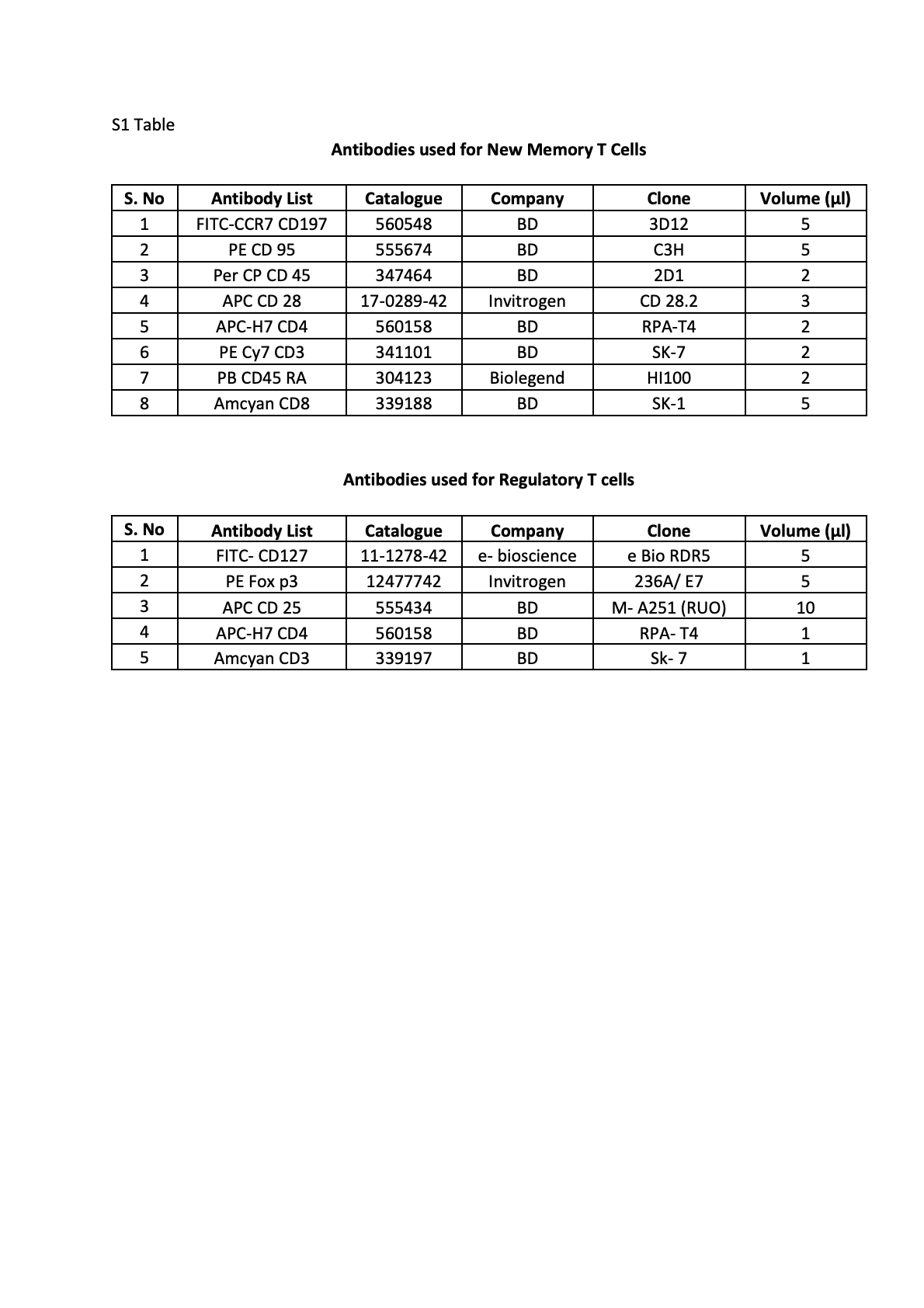

Supplement: S1 Table — (TIFF) [file pone.0258743.s002.tiff]
